# Peace-Making in Marsupials: The First Study in the Red-Necked Wallaby (*Macropus rufogriseus*)

**DOI:** 10.1371/journal.pone.0086859

**Published:** 2014-01-29

**Authors:** Giada Cordoni, Ivan Norscia

**Affiliations:** Museo di Storia Naturale - University of Pisa, Calci (Pisa), Italy; Institut Pluridisciplinaire Hubert Curien, France

## Abstract

The issue of reconciliation has been widely investigated in many eutherian mammal species. Nevertheless, no data are available for marsupial mammals. Indeed, the majority of reports focus on group dynamics from an ecological and reproductive perspective, but no study has investigated them from a social point of view. We observed the red-necked wallaby colony (*Macropus rufogriseus*) hosted at the Tierparc Zoo Berlin (Germany) and collected data on aggressive and post-conflict interactions between group members. We found that the phenomenon of reconciliation is present in the study species (mean group CCT 27.40% ±8.89% SE). Therefore, we demonstrated, for the first time, the occurrence of reconciliation in a gregarious marsupial mammal. Post-conflict reunion was not affected by the relationship quality between individuals (friendship or kinship) but it was fine-tuned according to the aggression intensity. For example, low intensity conflicts were reconciled whereas high intensity ones were not. Reconciliation reduced anxiety-related scratching in both of the former opponents and limited further attacks towards the victim during the post-conflict period. These findings suggest that the red-necked wallaby, like many eutherian species, can evaluate the costs of reconciliation and engage in peace-making behavior in the right contexts, in order to maximize its pay-offs.

## Introduction

Based on recent molecular and phylogenomic datasets [1]–[3], marsupials diverged from placental mammals 168–178 Mya (Early-Middle Jurassic). Marsupials occupy a variety of niches (terrestrial, arboreal, and aquatic environments) and adopt many different lifestyles. Indeed, some species are highly social whereas others are solitary. Additionally, marsupials can be carnivorous, herbivorous, or omnivorous [4]. For each of these life styles, marsupials have evolved a wide array of morphological, behavioral, and neocortical specializations that are strikingly comparable to those observed in eutherian mammals occupying similar niches [4], [5].

It is therefore expected that evolution has led mammals to develop similar solutions to face similar environmental challenges, such as group living in social or gregarious species [6].

An important behavioral phenomenon that allows individuals to coexist in cohesive groups and prevent social disruption is reconciliation [7]. The occurrence of reconciliation - defined as the first exchange of affinitive contact between opponents soon after a conflict [8] - has been demonstrated as a widespread phenomenon across social, placental mammals (e.g. domestic goats [9], horses [10], spotted hyenas [11], wolves [12], domestic dogs [13], dolphins [14], primates [15], [16]).

Here we investigated, for the first time, the phenomenon of reconciliation in a gregarious marsupial mammal belonging to the family Macropodidae, the red-necked wallaby (*Macropus rufogriseus*).

The red-necked wallaby lives in relatively small groups (10–30 individuals) [17], [18]. Males disperse from their natal home range when they reach sexual maturity (at around two years of age) whereas females may remain in their mother’s home ranges and form matrilineal associations. Different studies have pointed out that wallaby males spend more time than expected with other males, especially with individuals of comparable body size [17]–[20].

Even if groups may vary seasonally in size and composition (e.g. variation in age or sex ratio), red-necked wallabies form groups similar to those of other gregarious, herbivorous mammals [18], [21]. Moreover, wallabies can engage in intraspecific interactions, establishing social relationships with particular conspecifics [22]. For example, adult females - which regularly form aggregates - can coordinate their behaviour through visual and olfactory interactions [23]. They can also signal their reciprocal spatial position within the group via postures and gestures that are often very subtle [23]. Feeding areas are frequently shared by matrilineal relatives: the more time two wallabies spend together the greater is their tendency to share resources and to socially interact [18]. Moreover, males often engage in play fighting with peers and younger partners [24], [25]. There is some evidence suggesting a possible connection between the rates of play among males and the proportion of time they spend in close proximity and in engaging in affinitive interactions [25], [26].

Contact between group members includes aggression, which can affect, both directly and indirectly, the social status of individuals by disrupting the usual pattern of interaction and by jeopardizing the benefits associated with a particular social relationship [15], [27], [28].

As already reported above, in placental mammals, reconciliation is considered a behavioral strategy used to repair the loss of paybacks associated with social relationships [7], [15], [29]. Similarly, social relationships in gregarious marsupial species can also be negatively affected by the conflict. Thus, we expect to find the occurrence of reconciliation in red-necked wallaby (Prediction 1).

The Valuable Relationship Hypothesis predicts that reconciliation should be observed more often after conflicts between dyads sharing good relationships and/or kin [30]–[33]. This hypothesis has been confirmed in many eutherian mammal species (e.g. domestic goats [9]; bonobos [34]; lowland gorillas [35]; bonnet macaques [36]; domestic dogs [13]; wolves [12]; chimpanzees [37]). If this hypothesis also applies to marsupials, we expect to find the highest reconciliation levels between kin and/or strongly bonded wallabies. (Prediction 2).

The aftermath of a conflict is a highly unsafe period for the opponents, especially for the victim. In fact, the aggression may flare up again or other group members could re-attack the victim[38]–[40].

High intensity conflicts imply strong physical contact between opponents compared to low intensity attacks and in the post-conflict period severe conflicts can increase the social tension and the risk for the victim to be re-attacked [37], [41]. Therefore, we expect reconciliation to occur less frequently after high than after low intensity conflicts (Prediction 3).

In order for post-conflict reunion to occur, reconciliation must provide the opponents with benefits that outweigh costs. Many authors agree that reconciliation reduces the probability of further attacks, particularly towards the victim [38], [39], [41]–[43] and limits anxiety in the opponents [40], [44]–[48]. In red-necked wallaby groups, as in other mammals, individuals have to preserve at least the compatibility among conspecifics, which is based on the general tenor of social interactions and on the degree of tolerance between partners [49]. As suggested by Cords and Aureli [49], compatible group members may be more motivated to reconcile because there is a lower risk of renewed aggression and the cost of the conciliatory contacts can be reduced. Consequently, we expect to find a decrease of renewed aggression towards the victim after reconciled compared to non-reconciled conflicts (Prediction 4).

Placental and marsupial mammals have a similar basic physiology (vasopressin/oxytocin, or vasopressin/oxytocin-like neuropeptides) linked to the modulation of stress responses and anxiety (a proxy for stress) [50]–[53]. From mice to humans, self-scratching (hereafter scratching) appears to be one of the most reliable behavioral tools to measure anxiety. Indeed, anxiety states can share common biochemical origins with the physiological sensation of pruritus [54], leading to the itch-scratch cycle [55]–[57]. Mood manipulation via anxiolytic substances in New and Old World monkeys lead to the reduction of scratching rates (*Homo sapiens* [58], [59], *Macaca* spp [60], [61], *Callithrix* spp [62], [63]). Scratching has been found to increase in stressful social situations in many primate species, including humans (*Homo sapiens* [57], [64], [65], [66], *Pan troglodytes* [31], [67], *Gorilla gorilla gorilla* [68], *Papio Anubis* [69], lemurs [70], [71]). Specifically, scratching is influenced by the presence of conflicts [38], [40], [44], [45], [60], [69], [72] and the perceived risk of attacks in the social group [68].

Based on this framework we expect scratching in red-neck wallabies to increase after an aggressive event and decrease to baseline levels after post-conflict reunion, if reconciliation acts as an anxiety reliever (Prediction 5).

Overall, in this study we aimed at assessing the occurrence of reconciliation in the red-necked wallaby, by focusing on the factors (i.e. relationship quality, conflict intensity) that can influence this phenomenon and by evaluating its possible benefits. Our findings were compared and contrasted to the results obtained from studies on placental mammals to highlight similarities and differences.

## Materials and Methods

### Ethics Statement

The necessity of approval by University of Pisa, Italy was waived because this is a purely observation study. We did not have any kind of contact with animals; indeed, we observed wallabies out of the enclosures by recording their behaviours. Therefore, no specific permissions were required for these locations and activities, because people normally visit the park and the study did not involve manipulation of animals or vertebrate work/sacrifice/experiment. The Director of the Tierpark Zoo Berlin gave us the permission to conduct the research in the Park.

### Study Group and Data Collection

The present study was carried out in October-November 2008 on the colony of red-necked wallabies (*Macropus rufogriseus*) hosted at the Tierparc Zoo (Berlin, Germany) and composed of 16 individuals (10 adult and 6 immature individuals; Table 1). The animals were housed in a natural grass lawn enclosure of about 0.1 ha enriched with trees and branches. During the night, the subjects could freely move to and from an indoor facility. The wallabies received vegetables twice a day (8.30 a.m and 12.30 p.m.) and spent most of their time budget resting or foraging on leaves and branches always available in the enclosure. No stereotypic or aberrant behaviors were observed in this group.

**Table 1:** The composition of red-necked wallaby colony hosted at the Tierparc Zoo Berlin.

INDIVIDUAL	SEX	BIRTH DATE	RELATEDNESS
**K22**	M	1999	
**K10**	F	2008	
**K20**	F	2005	
**K8**	F	2002	
**K14**	M	2004	
**K1**	F	2001	
**K12**	M	2008	K1’s son
**K5**	F	2005	K1’s daughter
**K23**	F	2008	K5’s daughter
**K4**	F	2002	
**K24**	M	2008	K4’s son
**K11**	F	2003	K4’s daughter
**K2**	F	2005	K11’s daughter
**K9**	M	2008	K11’s son
**K3**	F	2006	K11’s daughter
**K21**	M	2008	K3’s son

We followed the group spanning early morning (06.00–08.00 a.m.) and evening (04.00–07.00 p.m.). We collected a total of 90 hrs of observation via the all occurrences sampling method [73]. The observation period included the maximum daytime activity of wallabies [74]. Individual recognition was possible due to external features and differential ear notching. Proper data collection started after a preliminary phase of 10 hrs, after checking that the observations by the two observers matched in at least 95% of cases [75]. All aggressive interactions between wallabies were collected live. For each interaction we noted: i) opponent identity, ii) context (feeding/foraging, social interaction, and resting); iii) type of conflict (decided, when the winner and loser could be clearly identified, or undecided); iv) aggressive behavioral patterns (see Table 2) and, v) conflict intensity. We defined two stages of conflict intensity: stage 1 (low intensity) - aggression without physical contact; stage 2 (high intensity) - aggression with physical contact. Moreover, we defined renewed aggression as an aggressive behaviour that the former aggressor directed to the same victim in the two minutes following the previous conflict [76].

**Table 2:** Aggressive and affinitive behavioural patterns [18]–[20], [24], [26] recorded in the red-necked wallaby group during the observation period.

PATTERN	DESCRIPTION
**Aggressive patterns**	
**Bite**	An individual bites aggressively a conspecific’s body part
**Chase**	An individual chases a conspecific by jumping rapidly behind him/her
**Dismiss**	An individual performs a brusque movement to keep away a conspecific
**Jump**	An individual aggressively jumps with the legs on conspecific’s body
**Kick**	An individual aggressively kicks a conspecific with both legs
**Punch**	An individual aggressively punches a conspecific’s face
**Push**	An individual aggressively pushes away a conspecific with the forelegs
**Affinitive patterns**	
**Sit in contact**	Two or more individuals sit with some parts of their bodies in contact
**Feed in contact**	Two or more individuals feed with some parts of their bodies in contact
**Food sharing**	An individual shares his/her food with a conspecific or permits a conspecific to take his/her food
**Grooming**	An individual cleans the fur of a conspecific with the mouth
**Play**	Two or more individuals engage in motor patterns (e.g. bite, chase) typical of ‘serious’ functional contexts but in a different manner. In fact, playful patterns are often exaggerated, reordered, incomplete,brief, repeated, varied in sequence and inhibited
**Social lick**	An individual licks a conspecific’s body part excluding ano-genital area
**Social sniff**	An individual sniffs a conspecific’s body part excluding ano-genital area

After the last aggressive pattern of any given agonistic event, we observed the victim as the focal individual for a ten-min Post-Conflict period (PC). Control observation (MC) took place in a following day at the same conditions as the original PC, on the same focal animal, in the absence of any agonistic interactions during the 10 min before the beginning of MC and when the opponents had the opportunity to interact [30], [77]. For both PCs and MCs we recorded: i) starting time (minute), ii) type of first affinitive interaction (feeding/sitting in contact, grooming, food-sharing, social licking/sniffing and playing; see Table 2), iii) the minute of first affinitive interaction, and iv) the initiator of the affinitive behaviour (the animal involved in a previous aggression - victim or aggressor - which first initiated an affinitive post-conflict interaction with the other opponent).

Some conflicts occurred during social interactions (such as social sniff/lick, foraging, and play). After a conflict, the two opponents normally separated from one another. However, the cases in which the animals remained in close proximity after a conflict were not included in the analyses.

### Data Analysis

For each victim we determined the number of attracted (A), dispersed (D), and neutral (N) pairs, over all PC-MC pairs (see Table 3). In attracted pairs, affinitive contacts occurred earlier in the PC than in the MC (or they occurred in the PC, but not in the MC). In dispersed pairs, affinitive contacts occurred earlier in the MC than in the PC (or they did not occur at all in the PC). In neutral pairs, affinitive contacts did not occur or occurred during the same minute in the PC and in the MC. The minimum number of PC-MC pairs per individual was set at 3. Individuals involved in less than 3 PC-MC pairs were removed from the analyses.

**Table 3:** The number of attracted (A), dispersed (D), and neutral (N) pairs per individuals.

INDIVIDUALS	A	D	N
**k22**	1	0	6
**k14**	2	0	2
**k1**	1	2	2
**k4**	0	2	2
**k8**	3	0	1
**k11**	1	0	3
**k2**	0	0	5
**k5**	2	0	3
**k20**	2	0	3
**k3**	4	0	0
**k9**	0	0	6
**k10**	6	2	6
**k12**	7	1	4
**k23**	4	1	3
**k24**	4	1	9
**k21**	6	2	7

To evaluate individual reconciliation, we used Veenema et al.’s [78] measure of Conciliatory Tendency (CCT), defined as “*attracted minus dispersed pairs divided by the total number of PC-MC pairs*”. Individual CCTs were used to determine the mean group CCT.

We evaluated the relationship quality between group members by measuring the baseline levels of affinitive contacts via all occurrences sampling method (see Table 2). The baseline levels were assessed by excluding PCs and MCs periods. To investigate the influence of relationship quality on reconciliation, for each individual we first calculated the mean value of the frequencies of the affinitive interactions for the dyads including the selected individual. Then, we divided the dyads including the selected individuals into two categories: weak and close dyads. The categories were assigned using the following criteria: the dyads whose affinitive contact frequencies were higher than the median value of the selected individual were labeled as “close” whereas the dyads whose affinitive contact frequencies were lower than the median value of the selected animal were labeled as “weak”.

All the analyses were carried out at the individual level. Due to the small sample size (8≤N≤16) and/or deviation from normality (Kolmogorov-Smirnov, p<0.05), and the fact that data were collected on a single group in a single period, we employed nonparametric statistical tests [79]. We made use of exact tests according to the threshold values indicated by Mundry & Fischer [80]. The post-hoc Dunnett’s multiple comparison test was used [79]. Statistical analyses were performed by using SPSS 19.0. We applied the Bonferroni correction according to the number of tests run on the same set of data. Owing to the fact that the Bonferroni method is concerned with the general null hypothesis (with all null hypotheses true simultaneously) and due to the increase in the likelihood of type II errors (“false negative”) as the number of comparisons increase, the discussion was also based on result significance and consistency [81]–[82].

The data file used to carry out the analyses performed in this study can be made freely available upon request.

## Results

### Occurrence of Reconciliation

**Figure 1:**
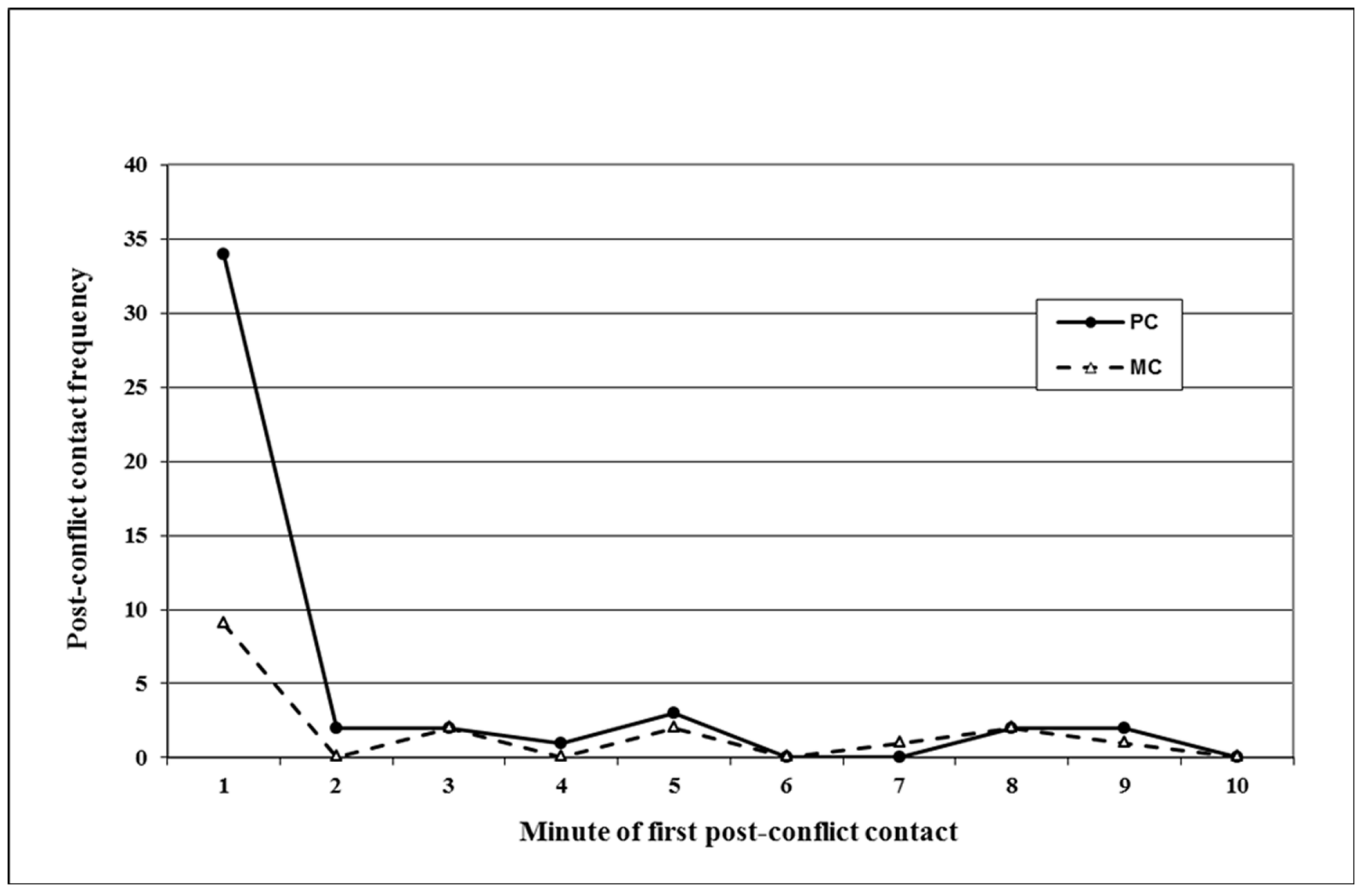
Temporal distribution of first conciliatory contacts in PCs (black circles) and MCs (empty triangles).

We collected 115 PC-MC pairs. The analysis revealed that the attracted pairs were significantly more frequent than the dispersed pairs (Bonferroni’s correction α = 0.025, Wilcoxon test: T = 10, ties = 2, N = 16, p = 0.005). The mean group CCT was 27.40% ±8.89% SE. As showed in the Figure 1, the majority of first affinitive contacts occurred within the first 2 minutes.

Both victims and aggressors initiated the first post-conflict affinitive contact with comparable frequencies (Wilcoxon test: T = 59.5, ties = 1, N = 16, p = 0.990).

The attracted pairs remained more frequent than the dispersed pairs even when restricting the analyses to the conflicts risen during social interactions (social lick/sniff and play) (Wilcoxon test: T = 0, ties = 2, N = 8, p = 0.031). For conflicts over food, there were no differences between attracted and dispersed pairs (Wilcoxon test: T = 0, ties = 1, N = 6, p = 0.063). The sample size for these analyses was reduced because we included only the individuals had at least 3 PC-MC pairs collected in social context.

### Reconciliation and Relationship Quality

We evaluated, at the individual level, the occurrence of reconciliation both in related (mother-offspring and siblings) and unrelated animals. Considering the related individuals present in the study group (N = 10), we did not obtain any statistical difference between attracted and dispersed pairs (Bonferroni’s correction α = 0.025, Wilcoxon test: T = 4, ties = 3, N = 10, p = 0.110). Instead, attracted pairs were significantly more frequent than dispersed pairs for unrelated individuals included in at least 3 PC-MC pairs (Wilcoxon test: T = 2, ties = 1, N = 15, p = 0.000). The unrelated animals for which it was possible to calculate the mean CCT for both weak and close relationships (N = 14) were included in the analysis to check for the influence of the relationship quality on post-conflict reunion. We found no significant difference in the CCT values between weakly and closely bonded individuals (Wilcoxon test: T = 45.5, ties = 0, N = 14, p = 0.682).

### Reconciliation and Conflict Intensity

Comparing the attracted and dispersed pairs in high intensity conflicts, we did not obtain any statistical difference (Bonferroni’s correction α = 0.025, Wilcoxon test: T = 4, ties = 6, N = 10, p = 1.000). Conversely, the analysis revealed that the attracted pairs were significantly more frequent than the dispersed pairs for low intensity conflicts (Bonferroni’s correction α = 0.025, Wilcoxon test: T = 6.5, ties = 3, N = 16, p = 0.004).

### Reconciliation and Scratching Levels

Following the consolidated method proposed by de Waal and van Roosmalen [8] we followed the victim of an aggression during PCs and MCs. Moreover, we recorded all scratching bouts via the all occurrence sampling method for both opponents in three different conditions: post-conflict period with no conciliatory contact between opponents (PCno), post-conflict period with conciliatory contact between opponents (PCyes), control condition with no previous aggression (MC).

**Figure 2:**
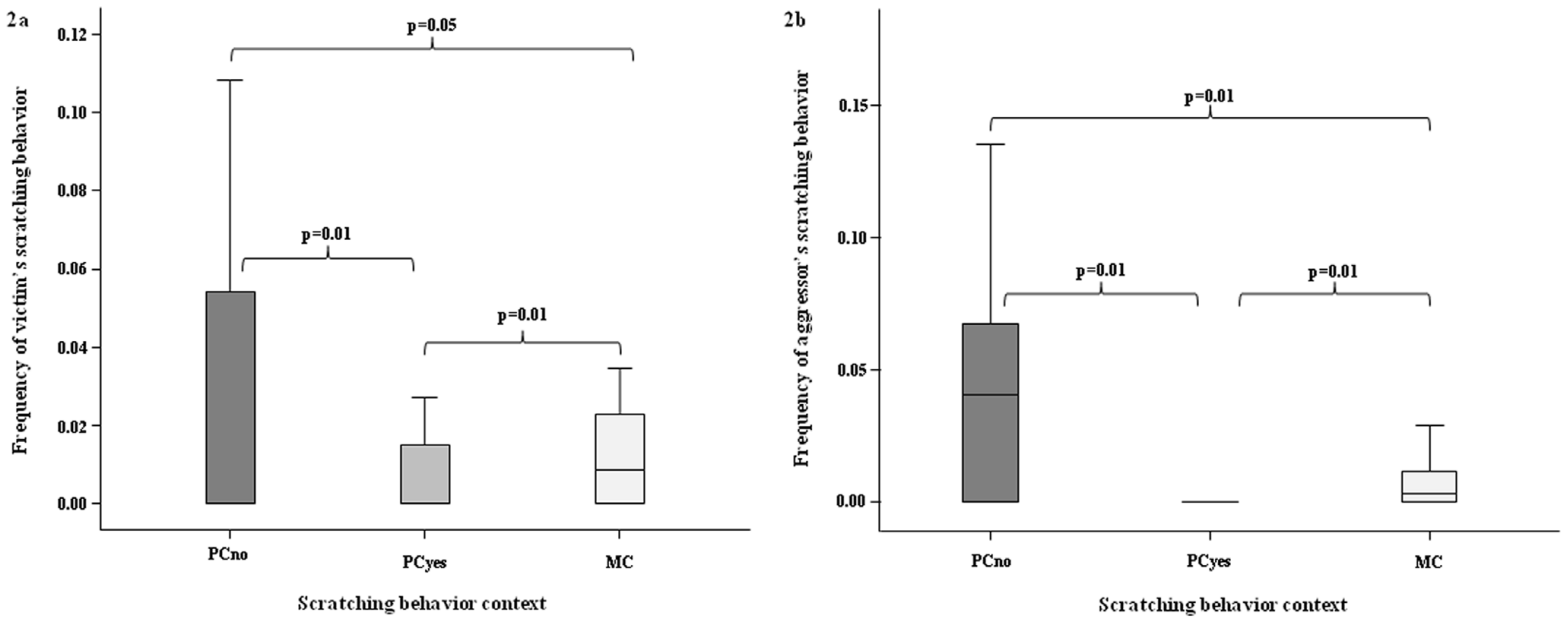
Scratching hourly frequency during post-conflict period with no reconciliation (PCno), post-conflict period with reconciliation (PCyes), and control condition (MC) both in the victim (2a) and in the aggressor (2b). Solid horizontal lines indicate medians; length of the boxes corresponds to inter-quartile range; thin horizontal lines indicate range of observed values (minimum and maximum). Only significant results are reported.

Considering the victim, a significant difference in the scratching levels was detected among the three diverse conditions (Friedman test: χ^2^ = 8.419, d.f. = 2, N = 16, p = 0.012) and between each pair of conditions. In particular, scratching increased after aggression and decreased to the baseline level after post-conflict reunion (Dunnet’s post-hoc test: scr_PCno_ *vs* scr_PCyes_ q = 2.032, p = 0.050; scr_PCno_ *vs* scr_MC_ q = 5.450, p = 0.010; scr_PCyes_ *vs* scr_MC_ q = 7.480, p = 0.010) (Figure 2a).

Considering the aggressor, we obtained the same results: a significant difference in scratching levels was detected among the three diverse conditions (Friedman test: χ^2^ = 9.256, d.f. = 2, N = 16, p = 0.007) and between each pair of conditions (Figure 2b). Particularly, scratching increased after aggression and decreased, to the baseline level, after post-conflict reunion (Dunnet’s post-hoc test: scr_PCno_ *vs* scr_PCyes_ q = 8.130, p = 0.010; scr_PCno_ *vs* scr_MC_ q = 3.230, p = 0.010; scr_PCyes_ *vs* scr_MC_ q = 4.90, p = 0.010).

### Reconciliation and Renewed Aggression

We evaluated if the occurrence of reconciliation affected the frequency of renewed aggression towards the victim of a former attack. The levels of renewed aggression were significantly higher in the absence of reconciliation than in its presence (Wilcoxon exact test: T = 3.5, ties = 0, N = 9, p = 0.027).

## Discussion

This study reveals, for the first time, the occurrence of reconciliation in a gregarious marsupial mammal, the red-necked wallaby (Prediction 1 confirmed). The relationship quality does not affect reconciliation rates either between kin or between dyads sharing close relationships (Prediction 2 not confirmed). Conversely, high intensity conflicts reduce the probability of post-conflict reunion (Prediction 3 confirmed). Finally, reconciliation decreases the probability of further attacks towards the victim during the post-conflict period (Prediction 4 confirmed) and can reduce the post-conflict scratching, linked to anxiety, in both the victim and the aggressor (Prediction 5 confirmed).

Red-necked wallabies can spend a considerable portion of their time budget ranging, foraging, or resting alone [18], [19]. However, they can also engage in social interactions via context-dependent reconciliation as it has been observed in placental mammals [7], [10], [12]. Wallabies reconciled only after low intensity aggression, associated with a lower risk of renewed attacks, thus suggesting that the individuals may evaluate the potential danger of reuniting with a former opponent before engaging in post-conflict affinitive contacts. The influence of conflict intensity upon the frequency of reconciliation has produced contradictory results in different studies. For example, in *Canis lupus* post-conflict reunions occur with comparable levels after both severe and mild aggressions [12]. Similarly, in hand-raised ravens the intensity of the conflicts did not affect the occurrence of reconciliation [83]. *Propithecus verreauxi*, a group-living lemur of Madagascar, engaged in conciliatory contacts after mild conflicts but not after severe ones [41]. In *Cebus capucinus* there was no significant difference in the probability of reconciliation according to conflict intensity [84]. In bonnet macaques (*Macaca radiata*), aggressions with physical contact were reconciled about two times more frequently than aggressions without contact [85]. Similarly, Cordoni and colleagues [35] found that lowland gorillas (*Gorilla gorilla gorilla*) reconciled only severe attacks. According to the results presented above, it seems that individuals engage in post-conflict reunion, regardless of conflict intensity [7], when reconciliation is crucial to maintain the group cohesion, necessary for individual survival, as in the case of wolves and gorillas [12], [35]. Wallabies, which can show gregariousness but do not base their subsistence primarily on social cohesion, can “afford” to decide to reconcile - if the conflict is mild - or to disperse - if the severity of a conflict makes any attempt to affiliate with the former opponent too risky.

Our findings also show that conciliatory contacts reduce the rate of renewed aggression towards the victim. A similar result was also obtained in great apes [37], [47], [86], [87] and two prosimian species, *Propithecus verreauxi* [41] and *Lemur catta* [88], which showed a reduction of further attacks towards the victim following a conciliatory contact. Conversely, in bonnet macaques (*Macaca radiata*), Cooper and colleagues [36] did not detect a decrease of renewed aggression received by the former opponent after reconciled conflicts. The authors suggested that post-conflict reunion did not fully restore and repair the relationship between opponents.

In our study, we found that both opponents experienced a decrease in scratching behaviour after a conflict was reconciled, thus suggesting that reconciliation works as an anxiety reliever. By reducing anxiety, reconciliation may limit the level of animal alertness towards renewed aggression and uncertain social situations [31], [38], [39], [43], [45], [46], [89]. This finding may suggest that placentals and marsupials employ similar behavioral solutions when facing comparable social challenges.

Compared to placental herbivorous, macropods do not show frequent overt social interactions (e.g. grooming, vigorous play). However, they perform continuous “covert” interactions (e.g. social sniffing, social licking, feeding in contact, scent marking) to determine and maintain their relative spatial positions and inter-individual associations [21], [23], [90]–[91]. Hence, in the red-necked wallaby groups the temporary lack of partner compatibility [49] after an aggressive encounter may jeopardize the normal social interactions and the degree of inter-individual tolerance both in the aggressor and in the victim. Reconciliation may represent a useful tool to restore relaxed social conditions. This hypothesis is supported by the fact that both victims and aggressors initiated conciliatory contacts with comparable frequency.

In red-necked wallabies the reconciliation does not follow the Valuable Relationship Hypothesis [31], [33]. In fact, kinship and relationship quality did not affect the conciliatory contact levels. This may be due to the fact that social bonding is not relevant to individual survival as in other placental mammals and kin relationship is not valued. In this respect, related males leave their natal groups and females show weak maternal care (mothers abandon their offspring once they leave the pouch and can eject the pouch-young when pursued by a predator) [91], [92]. Conversely, post-conflict reunions may be useful to preserve the compatibility with unrelated conspecifics (independently of the relationship quality) functioning as a shelter against retaliations or to co-exist peacefully when sharing feeding sites. This is in line with the fact that wallabies form occasional groups, with no permanent composition and different individuals, depending on the feeding site and the time [92].

In conclusion, as many eutherian species, red-necked wallabies may evaluate the costs of reconciliation, in order to maximize its pay-offs, such as the reduction of further attacks and the decrease of post-conflict anxiety. Further behavioral studies comparing metatherian and eutherian species, and more investigation on marsupial mammals, are necessary to explore convergent and divergent adaptations of this vertebrate class, and make inferences on the evolutionary roots of post-conflict behavior in mammals, including non-human primates and *Homo sapiens*.
